# Quantifying the health benefits of chronic disease prevention: a fresh approach using cardiovascular disease as an example

**DOI:** 10.1007/s10654-014-9932-1

**Published:** 2014-07-26

**Authors:** Nicholas J. Wald, Joan K. Morris

**Affiliations:** Wolfson Institute of Preventive Medicine, Barts and The London School of Medicine and Dentistry, Queen Mary University of London, Charterhouse Square, London, EC1M 6BQ UK

**Keywords:** Relative risk reduction, Absolute risk reduction, Health benefits, Polypill, Statins, Blood pressure lowering drugs, Salt intake reduction

## Abstract

Current methods of determining the proportion of people who benefit from a preventive intervention and the years of life gained can underestimate the former and overestimate the latter. We describe how to overcome these errors, using two examples relating to the prevention of myocardial infarction (MI) and stroke, one using a specified polypill daily from age 50 and another reducing salt intake in the population. Standard life table analysis was used to calculate the person-years of life gained without an MI or stroke, based on estimates of the incidence of these disorders in England and Wales. The proportion of individuals who benefit was taken as everyone who would, without treatment, have an MI or stroke (holistic model), rather than limiting the benefit to the proportion calculated from the relative risk reduction (reductionist model), as is current practice. Under the holistic model, 33 % of people who take the polypill from age 50 benefit, gaining, on average, 8 years of life without an MI or stroke (19 % and 14 years under the reductionist model). Estimates for reducing salt intake by 6 g/day are 33 % and 2.8 years respectively under the holistic model (6 % and 16 years under the reductionist model). In the prevention of disorders such as stroke by reducing exposure to causal factors such as blood pressure, the use of a holistic model corrects the underestimation of the proportion of people who benefit and the overestimation of their years of life gained associated with current methods.

## Introduction

In spite of many publications on the effects of interventions to prevent chronic diseases, there is no satisfactory method of accurately estimating and expressing the resulting health benefits. The benefits are often presented as the relative and absolute risk reductions, but these two measures can give contradictory impressions of the size of the benefit. For example, a preventive intervention that reduces the risk of a disease by 70 % confers an absolute risk reduction of only 0.7 % a year if the prevalence of the disease without treatment were 1 %. The absolute risk reduction takes account of the background incidence but estimates vary according to the place, time, and the time interval over which the risk reduction is considered (e.g. per year, per 10 years, etc.), and for disorders that become more common with increasing age, the estimates will not be a simple multiple of the time interval.

The difficulties can be overcome by using standard life table methods to estimate the person-years of life gained without the clinical events health interventions are designed to prevent (see for example Franco et al. [1]). But there remains a problem in using such estimates to determine the proportion of people who benefit, and among these an estimate of the average years of life gained.

The standard method of calculating these two measures of benefit, which is appropriate when the preventive effect benefits only a proportion of people who would, in the absence of intervention, have been affected, is to use the relative risk reduction to separate, into two groups, the number of people who would have had a clinical event that the intervention is designed to prevent: one that experiences all the benefit, and another that experiences no benefit. For example, if, in 1,000 people, ten would have a clinical event in the absence of treatment, and the preventive treatment reduces risk by 50 %, the benefit is taken to be limited to five of the ten, while the other five have no benefit at all. This model (which we refer to as the reductionist model) is sometimes appropriate, for example, with the use of folic acid supplements before and during early pregnancy to prevent a neural tube defect. In this example the only babies who benefit are those in whom the defect was prevented. Not all babies who would have had a neural tube defect in the absence of taking a folic acid supplement benefit; those who have such a defect in spite of taking supplements receive no benefit.

The standard method that uses the reductionist model is, however, not appropriate for the prevention of a chronic disease, such as ischaemic heart disease, in which clinical events arise from the disease over time, and the preventive effect is expected to benefit everyone who would, in the absence of intervention, have been affected. In these circumstances the incidence of clinical events arising from the disease is reduced by reducing exposure to the causes of the disease. In expectation, the clinical events will be delayed in everyone who would have had an ischaemic heart disease event when not taking preventive treatment, some for short periods and others by longer. The benefit will not be restricted to those for whom the ischaemic heart disease event was completely prevented; it will also extend to those for whom the event was delayed. In our example above, in which ten people have a clinical event in the absence of treatment, in expectation, all ten would benefit by having their clinical event delayed as well as prevented altogether. This implies a different model, which we propose here and refer to as the holistic model. The total person-years of life gained is the same for both models, say 100; in the holistic model each person who benefits gains 10 years on average (100/10) but in the reductionist model it is 20 (100/5).

We here use two examples to illustrate the application of the holistic model in determining health benefits: (1) the prevention of myocardial infarction and stroke through taking a combination of blood pressure lowering drugs and a statin (polypill), and (2) the prevention of these disorders through reducing daily salt intake.

## Methods

Standard life table methods were used to estimate two specified health benefits, namely (1) the proportion of individuals who adopt a preventive intervention who will directly benefit from the intervention (Health Benefit_proportion_; HB_p_) over their lifetime (up to age 99 or prior death), and among these (2) the average years of life gained without the disorder or disorders that the intervention is designed to prevent (Health Benefit_average gain_; HB_ag_). At the end of each year of age a person could be: (1) alive without the specified disorder, or (2) alive or dead with the specified disorder, or (3) dead without the specified disorder. This takes into account so called “competing” causes of death. Over time, individuals can move from 1 to 2 or from 1 to 3, but not from 2 to 3, 2 to 1, nor 3 to 1. Separate sex specific life tables were constructed for people who do not receive the preventive intervention and for those who do.

For people who do not have the preventive intervention, the probability of moving from state 1 to 2 was the age-sex specific annual incidence of the first occurrence of the specified disorder (see “Appendix” for details), and the probability of moving from state 1 to 3 was the age-sex specific annual mortality from all causes, excluding the specified disorder [2].

For people who do have the preventive intervention, the probability of moving from state 1 to state 2 was the age-sex specific annual incidence of the first occurrence of the specified disorder multiplied by the age-sex specific relative risk reductions arising from the preventive intervention, and the probability of moving from state 1 to 3 was the same as for people who did not have the intervention.

Under the holistic model the number of people who benefit is all those who had the specified disorder in the no intervention group. Under the reductionist model only a proportion benefit, that proportion being the estimate of the relative risk reduction. Numerically, this is equivalent to the number of people who benefit, taken as those who have the specified disorder in the no intervention group minus those who have the specified disorder in the intervention group. Their average gain in disorder-free life is the total disorder-free years of life gained, divided by the number of people who benefit under each model. No discounting of the gain was adopted because in policy terms a year of life gained in people of a given age now should not be assigned a greater value than one in people of the same age in the future [3]. Also, the use of quality adjusted life years (QUALYs) gained does not arise because we consider years gained without a clinical event that the intervention is designed to prevent.

**Fig. 1:**
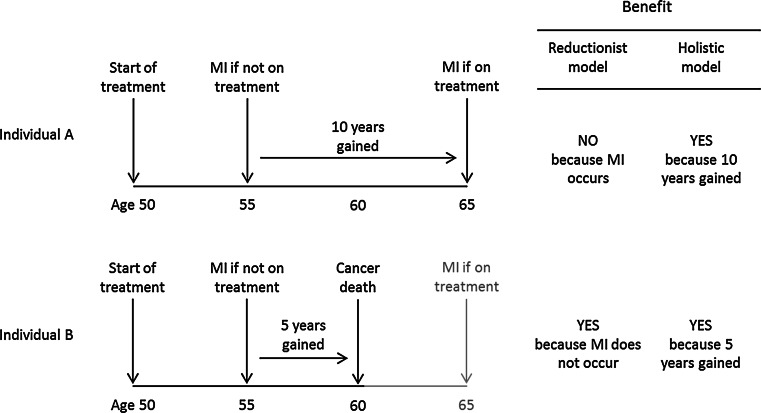
Illustration of the effect of intercurrent death on the classification of benefit under the reductionist and holistic models (*MI* myocardial infarction)

Figure 1 illustrates the difference between the holistic and reductionist models. The figure shows two hypothetical individuals (A and B) aged 50 who, without treatment, have a myocardial infarction at age 55, but on treatment this would have been delayed by 10 years. Individual A has a myocardial infarction aged 65, and Individual B dies of cancer at age 60. Under the holistic model both individuals benefit because both gain extra years of life without a myocardial infarction or stroke. A gains 10 years but B gains only 5 years. Under the reductionist model A does not benefit because he has a myocardial infarction, albeit 10 years later than would otherwise be the case, but B does benefit even though the benefit is less, due to the intercurrent death from cancer. The years of life gained for A and B together is 15, so under the reductionist model the average is 15 years (15/1) while under the holistic model it is 7.5 years (15/2). The reductionist model systematically underestimates the proportion of people who benefit and overestimates the years of life they gain.

We applied the holistic model to two different interventions, both designed to prevent a first myocardial infarction or stroke, and compared the results with those obtained using the reductionist model. We did not perform an economic analysis because the purpose of our paper was limited to assessing health benefits. The two interventions were: (1) use of a daily four component polypill consisting of amlodipine 2.5 mg, losartan 25 mg, hydrochlorothiazide 12.5 mg, and simvastatin 20 mg taken from ages 50, 60, 70 or 80 and (2) daily salt intake reductions of 1.5, 3, 4.5 and 6.0 g. We used these two examples because their effects are well documented quantitatively, and because of their potential in preventing cardiovascular disease [4–8]. We also considered the blood pressure drugs used alone, the statin alone, and both the salt reduction and polypill used together. The appendix gives further details specific to these examples.

Statistical analyses were performed using the R statistical package.

## Results

Table 1 shows estimates of the two specified health benefits (HB_p_ and HB_ag_) using the holistic model, according to the age at which people start to take the polypill. The table also shows the relative and absolute risk reductions. The relative risk reduction is 56 % in people starting treatment at age 50, and the absolute annual risk reduction is 0.37 %. An estimated 33 % of people taking the preventive treatment benefit (HB_p_), and these gain, on average, 8.0 years of life without a myocardial infarction or stroke (HB_ag_). The remaining 67 % do not benefit, because they die from another disorder before they would have had a myocardial infarction or stroke.

**Table 1:** Estimates relating to the prevention of a first myocardial infarction (MI) or stroke (“disorder”) in people taking the polypill daily from specified ages

Taking polypill from specified age to age 99	Proportion of people who will have first MI or stroke in the absence of treatment (%)	Proportion of people who will have first MI or stroke whilst taking polypill (%)	Relative risk reduction (%)	Absolute annual risk reduction (%)	Proportion who benefit (HBp) (%)	Among those who benefit : average years of life gained without an MI or stroke (HBag)
50	33	15	56	0.37	33	8.0
60	33	15	55	0.45	33	6.7
70	31	15	53	0.55	31	5.1
80	29	14	51	0.74	29	3.4

Table 1 shows that the specified health benefits diminish with increasing age of starting the polypill, both in respect of the percentage who benefit and the average years of life gained, although the effect on the percentage who benefit is modest. For example, at age 50 the percentage who benefit is 33 % compared with 29 % for those who start at age 80, and the years of life gained reduce from 8.0 to 3.4, respectively.

Table 2 shows estimates of the health benefits among individuals aged 50 or older according to the level of salt intake reduction. If salt intake is reduced by 6 g/day, 33 % of individuals benefit (HB_p_) and they gain, on average, 2.8 years without a myocardial infarction or stroke (HB_ag_).

**Table 2:** Estimates relating to the prevention of a first myocardial infarction (MI) or stroke in individuals aged 50 and above according to specified daily salt reduction

Salt reduction (g/day)	Proportion of people who will have first MI or stroke in the absence of treatment (%)	Proportion of people who will have first MI or stroke with a reduced salt intake (%)	Relative risk reduction (%)	Absolute annual risk reduction (%)	Proportion who benefit (HBp) (%)	Among those who benefit: average years of life gained without an MI or stroke (HB_ag_)
1.5	33	32	4	0.03	33	0.7
3.0	33	30	9	0.06	33	1.5
4.5	33	29	13	0.09	33	2.1
6.0	33	28	17	0.11	33	2.8

Table 3 compares the two specified health benefit measures among people aged 50 and over for different preventive strategies. The proportions of people who benefit are the same (33 %), but the years of life gained without a myocardial infarction or stroke varies from 2.8 to 8.8 years.

**Table 3:** The two measures of health benefit in people aged 50 and over according to different preventive interventions to reduce the risk of a first myocardial infarction (MI) or stroke

	Reducing salt by 6 g/day	Taking simvastatin 20 mg daily from age 50	Taking 3 blood pressure lowering drugs at half standard dose daily from age 50	Taking polypill daily from age 50 (all 4 drugs)	Reducing salt by 6 g/day and taking polypill daily from age 50
Proportion who benefit (HB_p_)	33 %	33 %	33 %	33 %	33 %
Among these: average years of life gained without an MI or stroke (HB_ag_)	2.8	3.9	5.4	8.0	8.8

## Discussion

Specifying both the percentage of people who benefit from a health intervention (HB_p_) and, among these, the average years of life gained without the disorder in question (myocardial infarction and stroke in our examples) is a simple, informative way of expressing benefits in preventive medicine. Our analysis shows the importance of determining whether a holistic or reductionist model is used to calculate these estimates.

The holistic model is appropriate when the preventive measures exhibit a continuous biological action, such as blood pressure reduction, in which everyone experiences a reduction and the health benefits are expected to accrue to everyone who would have had an event in the absence of preventive intervention by delaying the event as well as possibly avoiding it. Had the reductionist model been used, only an estimated 19 % (33 % minus 15 % from Table 1) of people aged 50 or over would benefit, but they would gain, on average, more years of event-free life—14 years instead of 8.0.

With a 6 g/day reduction in salt intake, using the holistic model estimates showed that 33 % of people benefit and gain an average of 2.8 years of life without a myocardial infarction or stroke. The corresponding figures using the reductionist model are 6 % and 16 years respectively.

Risk of a myocardial infarction or stroke is currently often estimated in terms of the probability that a person will develop a clinical event over the next 10 years, and a risk “threshold” (say a 20 % 10-year risk) is used to identify people for preventive treatment [9]. Giving a risk estimate for “the next 10 years” for a preventive treatment that is intended to be taken indefinitely will underestimate both the risk and the potential benefit, as most of the preventable events will arise after 10 years. We therefore used lifetime benefit, in which the relative risk reduction decreases with age and the absolute risk reduction increases. For example, the relative risk reduction from age 50–59 is 81 % (see Table 6 in the Appendix), and the absolute annual risk reduction is 0.21 % over this ten year period. At age 80–89 the relative risk reduction is 55 % and the corresponding absolute annual risk reduction is 1.0 %. Another problem with using risk to prompt intervention is that it is the size of the health benefit of the proposed treatment that is relevant, rather than the risk itself. It is the translation of the reduction in incidence rates into extended years of life that is important. Identifying a high risk group without an effective treatment is pointless. It is the final benefit that needs to be the basis for decision making, and the estimate of health benefit should be life-long, not time limited.

Regardless of whether, in a particular context, the reductionist or holistic model is appropriate, the two specified measures of health benefit overcome limitations associated with the use of relative and absolute risk reduction, but the latter are still needed to calculate the two specified measures of health benefit. Our estimate of the benefit is robust for two reasons. First the estimates of relative risk reduction come from the results of large cohort studies and many randomized trials that show considerable consistency between studies. Second, sensitivity analyses showed that estimation of the specified health benefits were robust to small changes in the estimates of relative risk reduction, with an approximate proportional relationship between relative risk reduction and years of life gained without a myocardial infarction or stroke. So, for example, a 5 % change in the relative risk reduction would result in about a 5 % change in years of life gained. Sensitivity analyses also showed that changes in the incidences of the disorders in question affect the percentage of people who benefit from preventive interventions to an approximately proportionate extent, so that, for example, doubling the incidence in our examples increases the proportion of people who benefit from 33 to 50 %, or from 1:2 to 2:2. However, among those who benefit, the gain in life without the specified disorders remains similar.

Sometimes the benefit from a health intervention is expressed as the number needed to treat (NNT), which is the inverse of the absolute risk reduction. The NNT defined in this way is valid under the reductionist model, but not under the holistic model. The benefit from a health intervention is also sometimes expressed as the years of life gained divided by the number of people who adopt the preventive intervention. This is misleading, because some people who adopt the intervention cannot possibly benefit, for example, a person who takes a statin and dies in a road traffic accident a month later or someone who simply stops treatment. Instead of estimating the benefit to everyone adopting the intervention, it is more informative to separately estimate the proportion of people who will benefit, and among them estimate the average years of life gained without a clinical event the treatment prevents.

If the age-specific incidence rate of serious adverse effects were known, these could be included in the life table analysis together with the incidence of myocardial infarction or stroke. The benefit is then the avoidance of all these outcomes rather than preventing a myocardial infarction or stroke only. In our examples, the issue is minor, because there is strong evidence that salt reduction and the components of the polypill are almost free from serious adverse effects, with the exception of the rare occurrence of statin induced rhabdomyolysis. If this were included as a hazard, neither the percentage of people who benefit nor the years of life gained would differ at the level of precision used here, because of the rarity of the adverse reaction. Current estimates suggest that statin therapy may increase the risk of clinical diabetes by about 9 % [10]. Our method allows for any increase in the risk of myocardial infarctions and stroke arising in this way, but not for other complications of diabetes.

In this paper we consider years of life gained without an incident myocardial infarction or stroke. The same method of analysis as that described here could be applied to the prevention of death from these disorders, in which case the proportion of people benefitting would be less as not everyone who has a myocardial infarction or stroke will die from these disorders, but the years of life gained would be greater due to the inclusion of years of life after a first clinical event, as well as years gained before such an event. We selected myocardial infarction and stroke since they are “hard” end points for which estimates of incidence are available. Had, for example, angina been included, the benefits would have been greater.

The approach we propose, which is based on using standard life-table methods could, to advantage, be readily adopted, relying on estimates of relative and absolute risk reductions and data on cause-specific mortality from national vital statistics. The calculations are straightforward. Life-table methods are often used in economic cost-benefit analyses, but less so in papers that assess only health benefits.

In summary, the health benefits of preventive interventions are usefully presented in terms of the proportion of people receiving an intervention who benefit from it and their average years of life gained. These two measures overcome the apparent contradictory impressions arising from reporting estimates of the absolute and relative risk reduction. In the prevention of chronic disease, where the biological actions of an intervention exhibit continuous effects, the two measures of health benefit, calculated using the holistic model, provide a simple and accurate summary of the impact of the intervention for individuals and for populations as a whole.

## Appendix

To assess the health benefits arising from the prevention of myocardial infarction and stroke through taking a polypill or through reducing salt intake the following data were used.

1. The incidence of first myocardial infarction or stroke in England and Wales in 2010 in people not taking statins or blood pressure drugs
2. The age specific relative risks of a stroke or myocardial infarction whilst on the polypill
3. The age-specific relative risks of a stroke or myocardial infarction due to salt reduction
4. The age-specific mortality from all causes other than myocardial infarctions or strokes in England and Wales 2010

These are estimated below in the correspondingly numbered sections.

## 1. The incidence of first myocardial infarction or stroke in England and Wales in 2010 in people not taking statins or blood pressure drugs

To estimate this, we first used published estimates of the incidence of these disorders in 1985–1995 (section headed “Annual incidence of first myocardial infarction (MI) and stroke from 1985–95 [11]”) and then adjusted them for the reductions in incidence that occurred between 1995 and 2010 (section headed “Allowing for the decrease in incidence from 1985–95 to 2010”), and then took account of the fact that about 30 % of people aged 50 and older were taking statins or blood pressure drugs in 2010 (section headed “Allowing for the current use of components of the polypill in 2010”).

### Annual incidence of first myocardial infarction (MI) and stroke from 1985 to 1995 [11]

The following unpublished weighted logistic regression equations from the meta-analysis reported by Law et al. [11] were used to obtain yearly age specific incidence rates for men:

- incidence of first MI = exp(−8.9041 + 0.06148 × years)/[1 + exp(−8.9041 + 0.06148 × years)], and
- incidence of first stroke = exp(−11.3454 + 0.08769 × years)/[1 + exp(−11.3454 + 0.08769 × years)].

For women:

- incidence of first MI = exp(−12.5712 + 0.10332 × years)/[1 + exp(−12.5712 + 0.10332 × years)], and
- incidence of first stroke = exp(−11.8133 + 0.09112 × years)/[1 + exp(−11.8133 + 0.09112*years)]

### Allowing for the decrease in incidence from 1985–95 to 2010

The above incidence estimates relate to 1985–1995. Since then mortality from myocardial infarction and stroke has decreased [ONS 1985–1995 [12] versus ONS 2010 [2]; column 3 in Table 4 (Appendix)] as a result of both a decrease in incidence and a decrease in case-fatality. Two studies [13, 14] reported the contributions to the decrease in mortality arising from a decrease in incidence (I) compared with a decrease in case fatality (CF) (column 4 in Table 4 (Appendix)). The decrease in incidence of first MI and strokes from 1985–95 to 2010 (column 5) was estimated using the results of these two studies and assuming I and CF changed, over time, by the same proportion (P) (so that the decrease in incidence is P × I and the decrease in case fatality is P × CF). Then the decrease in mortality is 1 – [(1 – P × I) (1 – P × CF)]. For example in the second row of Table 4 (Appendix) the decrease in mortality is 67 %, so 0.67 = 1 – (1 − 0.30P) (1 − 0.43P) which can be rearranged so that 0.129P^2^ − 0.73P + 0.67 = 0. This quadratic equation has two solutions; P = 1.15 and P = 4.51. P = 4.51 leads to a decrease in incidence >100 %, which is not possible, so the decrease in incidence, P × I = 1.15 × 30 % = 35 % (as given in col 5).

**Table 4:** Estimation of the decrease in incidence of first myocardial infarction (MI) and stroke from 1985–95 to 2010

Gender	Disorder	Observed decrease in mortality (%) from 1985–95 to 2010	Decrease in incidence compared with reduction in case fatality (%) [13, 14]	Estimated decrease in incidence (%) from 1985–95 to 2010
Female	MI	76	31:29	53
Female	Stroke	67	30:43	35
Male	MI	69	33:24	51
Male	Stroke	64	30:43	33

The age specific incidence of a first myocardial infarction and stroke in 2010 was estimated by multiplying the estimated decreases in incidence from 1985–95 to 2010 (column 5 in Table 4 (Appendix)) by the logistic regression equations for the age specific incidence of a first myocardial infarction and stroke in 1985–95 given in the section above headed "Annual incidence of first myocardial infarction (MI) and stroke from 1985 to 1995".

### Allowing for the current use of components of the polypill in 2010

Around 30 % of people aged 50–99 were currently taking blood pressure lowering drugs [15] or statins [16] in 2010. Therefore the estimated age specific incidence in 2010 at each age was adjusted by 1/(0.7 + 0.3 × age specific relative risk as given in Table 5 (Appendix)) to estimate the incidence in people not taking statins or blood pressure drugs. Details of the estimation of the age specific relative risks are given below.

**Table 5:** Age specific relative risk estimates

Age taking polypill	Relative risk of a first stroke on daily polypill^a^	Relative risk of a first myocardial infarction on daily polypill^a^
50	0.26	0.13
60	0.31	0.23
70	0.38	0.33
80	0.51	0.37
90+	0.51	0.37

^a^Polypill contained amlodipine 2.5 mg, losartan 25 mg, hydrochlorothiazide 12.5 mg and simvastatin 20 mg

## 2. Estimating the age specific relative risks of a stroke or myocardial infarction or stroke on the polypill

Table 5 (Appendix) shows the age specific relative risk of a first myocardial infarction or stroke based on three sources [4–7]. The age specific relative risk estimates by single year of age for people aged 50–90 were obtained by linear interpolation using the relative risks in Table 5 (Appendix). For people age 90 and above the relative risks were assumed to be constant.

The relative risk reduction on the polypill taken daily decreases with age (see Table 5 (Appendix)) and therefore the average relative risk reduction in people age 50 and over (56 % in Table 6 (Appendix)) is lower than in the 50–59 category (81 %), which is similar to the previously published estimate for a 6 component polypill taken from age 55 [4].

**Table 6:** Average relative risk reductions (%) of a first myocardial infarction or stroke according to age at starting polypill and years of follow-up

Years of follow up	Age starting to take polypill daily			
	50	60	70	80
10	81	74	64	55
20	75	67	57	51
30	68	59	53	–
40	60	55	–	–
50	56	–	–	–

## 3. Estimating the age specific relative risks of a first myocardial infarction or stroke due to salt intake reduction

Table 7 (Appendix) shows the fall in diastolic blood pressure and the age specific relative risk of a first myocardial infarction or stroke according to salt intake reduction based on Ref. [8]. The age specific relative risk estimates by single year of age for people aged 50–90 were obtained by linear interpolation using the relative risks in Table 7 (Appendix). For people age 90 and above the relative risks were assumed to be constant.

**Table 7:** Reduction in systolic blood pressure (SBP) and age specific relative risk estimates of a first myocardial infarct (MI) or stroke according to age and salt intake reduction

Age	1.5 g/day			3.0 g/day			4.5 g/day			6 g/day		
	SBP reduction in mmHg	Relative risk of a first MI	Relative risk of a first stroke	SBP reduction in mmHg	Relative risk of a first MI	Relative risk of a first stroke	SBP reduction in mmHg	Relative risk of a first MI	Relative risk of a first stroke	SBP reduction in mmHg	Relative risk of a first MI	Relative risk of a first stroke
45	1.7	0.87	0.83	3.3	0.76	0.68	4.9	0.66	0.56	6.6	0.58	0.47
55	2.3	0.85	0.76	4.6	0.72	0.58	6.9	0.61	0.44	9.2	0.52	0.34
65	2.6	0.85	0.77	5.2	0.72	0.60	7.7	0.61	0.46	10.3	0.52	0.35
75	2.7	0.87	0.80	5.4	0.75	0.65	8.1	0.65	0.52	10.8	0.57	0.42
85	2.7	0.90	0.87	5.5	0.81	0.76	8.2	0.72	0.66	11.0	0.65	0.57

## 4. Estimating the age-specific mortality from all causes other than a myocardial infarction or stroke in England and Wales in 2010

This was obtained from the ONS publication Mortality Statistics: Deaths Registered in England and Wales (Series DR), 2010 [17].
